# Supplementary material for: Matrisome AnalyzeR – a suite of tools to annotate and quantify ECM molecules in big datasets across organisms
Source: J Cell Sci. 2023 Sep 4;136(17):jcs261255. doi: 10.1242/jcs.261255 (PMC10499032; doi:10.1242/jcs.261255)
Supplement: Supplementary information [file joces-136-261255-s1.pdf]

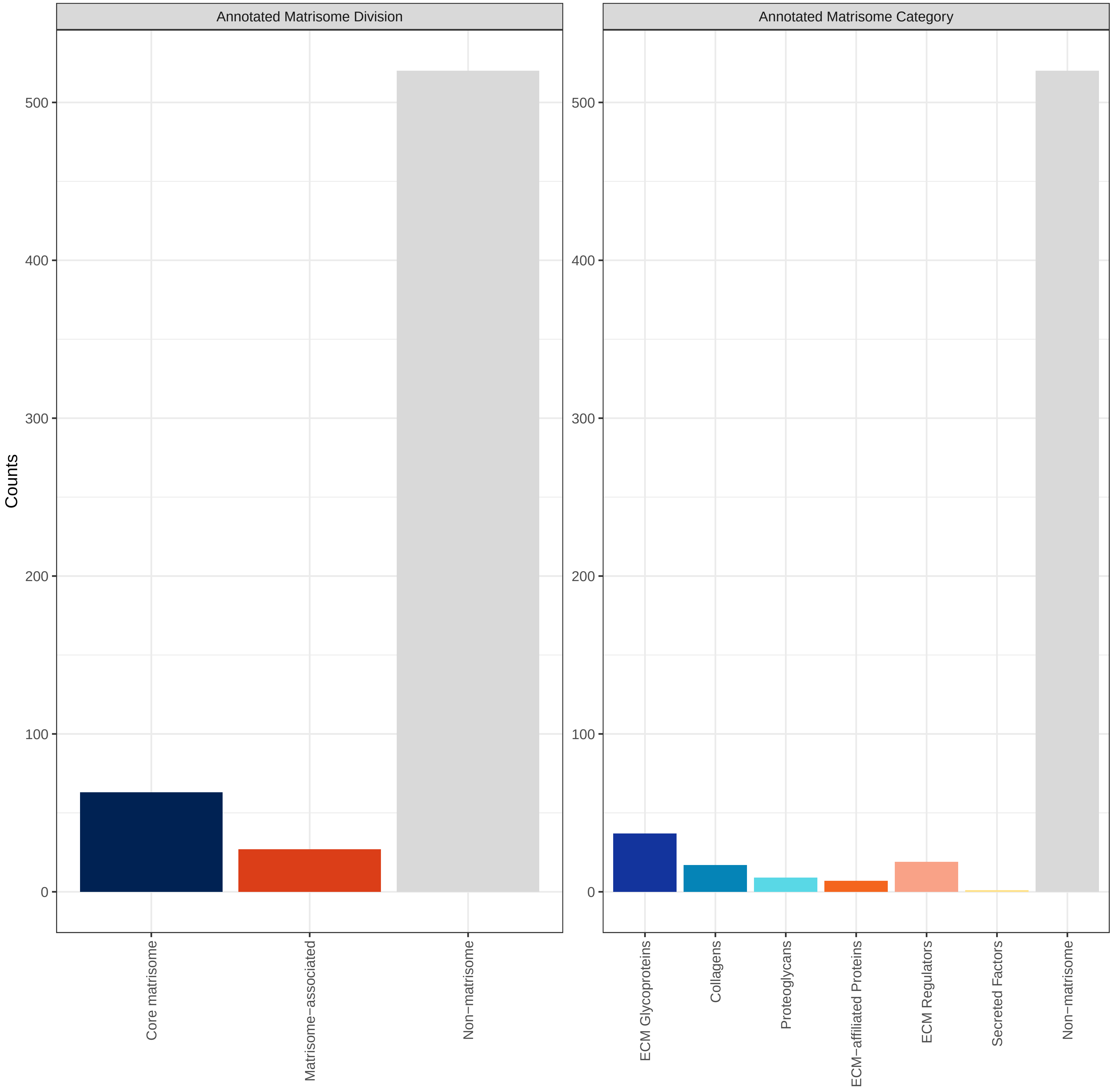

Fig. S1. Bar graphs resulting from inputting the test file provided as Supplementary File S1 and selecting the "Annotate" workflow of the web-based Matrisome AnalyzeR application.

Journal of Cell Science • Supplementary information

Table S1. Matrisome AnalyzeR_Test file
.csv file containing proteomic data from three technical replicates on ECM samples from human Fallopian tubes (Renner et al., 2022) used as an example to demonstrate the functionalities of the web-based Matrisome AnalyzeR application.

Click here to download Table S1

Table S2. .csv file resulting from inputting the test file provided as Table S1 and selecting the "Annotate" workflow of the web-based Matrisome AnalyzeR application.

Click here to download Table S2

Table S3. Analysis: .csv file resulting from inputting the test file provided as Table S1 and selecting the "Annotate & Analyze" workflow of the web-based Matrisome AnalyzeR application.

Click here to download Table S3
