# Supplementary material for: Matrisome AnalyzeR – a suite of tools to annotate and quantify ECM molecules in big datasets across organisms
Source: J Cell Sci. 2023 Sep 4;136(17):jcs261255. doi: 10.1242/jcs.261255 (PMC10499032; doi:10.1242/jcs.261255)
Supplement: Supplementary file 1 [file joces-136-261255_review_history.pdf]

Matrisome AnalyzeR: A suite of tools to annotate and quantify ECM molecules in big datasets across organisms

Petar B. Petrov, James M. Considine, Valerio Izzi and Alexandra Naba
DOI: 10.1242/jcs.261255

Editor: Kathleen Green

Review timeline

Original submission:	18 April 2023
Editorial decision:	9 June 2023
First revision received:	26 July 2023
Accepted:	1 August 2023

Original submission

First decision letter

MS ID#: JOCES/2023/261255

MS TITLE: Matrisome AnalyzeR: A suite of tools to annotate and quantify ECM molecules in big datasets across organisms

AUTHORS: Petar B Petrov, James M Considine, Valerio Izzi, and Alexandra Naba
ARTICLE TYPE: Tools and Resources

We have now reached a decision on the above manuscript.

To see the reviewers' reports and a copy of this decision letter, please go to: https://submit-jcs.biologists.org and click on the 'Manuscripts with Decisions' queue in the Author Area. (Corresponding author only has access to reviews.)

As you will see, the reviewers raise a number of substantial criticisms that prevent me from accepting the paper at this stage. They suggest, however, that a revised version might prove acceptable, if you can address their concerns. If you think that you can deal satisfactorily with the criticisms on revision, I would be pleased to see a revised manuscript. We would then return it to the reviewers.

Please ensure that you clearly highlight all changes made in the revised manuscript. Please avoid using 'Tracked changes' in Word files as these are lost in PDF conversion.

I should be grateful if you would also provide a point-by-point response detailing how you have dealt with the points raised by the reviewers in the 'Response to Reviewers' box. Please attend to all of the reviewers' comments. If you do not agree with any of their criticisms or suggestions please explain clearly why this is so.

Reviewer 1

Advance summary and potential significance to field

This paper describes a web tool that will allow researchers to identify which genes within a list encode known extracellular matrix proteins. This builds on their previous published work

© 2023. Published by The Company of Biologists under the terms of the Creative Commons Attribution License (https://creativecommons.org/licenses/by/4.0/).

assembling such lists of genes encoding ECM proteins from humans and a number of model organisms. It basically makes it easy to compare two lists, which one can already do using for example VLOOKUP in excel. They also include some visualization tools, but I did not find the diagrams in Figure 2 very illuminating. Table 1 was missing and the purpose of duplicating Figure 1B as Supplementary Figure S1 was not clear. This tool may be useful to some readers of JCS.

Comments for the author

The problem is that the vast majority of researcher will not only want to check whether their list of genes contains extracellular matrix proteins, and therefore will find more useful a general tool to indicate enrichment of any type of gene. This is commonly done with GO enrichment tools, as GO annotation of gene product function is an active and ongoing project for these organisms. Relevant to this, there is no mention of how the ECM protein/gene lists will be updated to incorporate new discoveries; without this the lists will progressively go stale. For these reasons, I doubt that there will be extensive use of this tool. I would urge the authors to work with HGNC, Uniprot, and the model organism databases to capture the information of the curated ECM gene lists they have produced via GO annotation and Gene Groups, as then the information will be accessible via diverse GO enrichment tools, and updated with new research.

<u>Reviewer 2</u>

Advance summary and potential significance to field

This work has the potential to be a successful tool for ECM research.

Comments for the author

The work is interesting and could be resourceful for the ECM community. However there are certain points that needed to be addressed by the authors:
1. What kind of MS quantitation would be utilised for input file? IS it spectral count or LFQ or MS1 based quant? It is not clear. This is important that in general, researchers uses many different open-source and vendor specific search engine based methods for quantitative Mass-Spectrometry.
2. It would be great if the authors can at least integrate the output files from the most popularly used quant tools such as Maxquant and Skyline (open source).
3. Authors should include in the input template regarding the quant criteria based on at least 2 or more unique peptides. Because, there could be multiple 1 peptide hits. For example, in the provided example data sheet- there are many isoforms of T-complex protein 1. How do the authors ensure the individual isoform protein group ID?
4. It is possible that, the data was acquired by a rigorous biochemical enrichment of ECM (following decellularization) followed by MS analysis still a number of cytoskeletal proteins were identified. It has been shown that cytoskeleton proteins could actually be connected with the ECM and most of the times they are indeed enriched with ECM proteins (example- Suleiman et.al eLife- Jeff Miner's work). How do the authors would annotate them? Comments and relevant modification on this aspect would be appreciated.
5. Could the authors built in a plug-in tool to prepare an abundance curve of the ECM proteins to estimate the distribution pattern?
6. If the authors aims to analyze based on relative quant then statistical tests should be incorporated with FDR correction option.

© 2023. Published by The Company of Biologists under the terms of the Creative Commons Attribution License (https://creativecommons.org/licenses/by/4.0/).

First revision

Author response to reviewers' comments

Reviewer 1

Advance Summary and Potential Significance to Field:

This paper describes a web tool that will allow researchers to identify which genes within a list encode known extracellular matrix proteins. This builds on their previous published work assembling such lists of genes encoding ECM proteins from humans and a number of model organisms. It basically makes it easy to compare two lists, which one can already do using for example VLOOKUP in excel. They also include some visualization tools, but I did not find the diagrams in Figure 2 very illuminating. Table 1 was missing and the purpose of duplicating Figure 1B as Supplementary Figure S1 was not clear. This tool may be useful to some readers of JCS.
> We thank the reviewer for this feedback and for finding that the tool "makes it easy to compare lists"
and that "This tool may be useful to some readers of JCS".

While Excel can certainly be a useful tool, we disagree that one can perform the same analysis permitted by Matrisome AnalyzeR "using VLOOKUP in Excel". We highlight here the most critical limitations of Excel and contrast them with the key functionalities of Matrisome AnalyzeR, hoping to demonstrate the usability and versatility of our web application:
1) Data format inconsistency: Excel sources the decimal/thousand format from the local system preferences, automatically deciding on the scope of commas, dots, slashes, dashes, and other symbols. Format misinterpretation is common when data are imported into Excel (*e.g.*, from US and EU scientists), and selecting specific data formats post-import does not guarantee the recovery of wrongly imported data formats. Additionally, data format operations in Excel are either file-wide or column-specific, making it largely impractical for files with mixed data types (*e.g.*, numbers and text, or numbers in different formats such as decimals and percentiles). Matrisome AnalyzeR uses nested heuristics to guarantee proper data formatting. Following proper data formatting prompt from the help box accessible by clicking the "?" button guarantees an error-free experience, yet the set of heuristics we implemented is largely able to handle different situations and, in our tests, managed to correctly parse data sheets ignoring any of the abovementioned suggestions.
2) File format restrictions: The NET framework integration within Excel guarantees software ability to access and, possibly, parse various file formats, including tabular and database ones. For example, there is no support for compressed R data formats (.RDS), which are widespread in the transcriptomics field, or for application-specific formats, such as the Skyline format for proteomics. While dedicated software exists for both formats mentioned above, a user willing to use them with Excel would *de facto* need to operate them first, export the data in a suitable new format, and then import them into Excel. Conversely, Matrisome AnalyzeR offers native support for both formats, meaning that they can simply be loaded as they are into the app and parsing will automatically start thanks to the heuristics (see above).
3) Size limit and operability. According to Microsoft (see Excel specifications and limits), the maximum number of rows in an entire workbook is approx. $1*10^6$ and the number of rows approx. $16*10^4$, though these limits are theoretical and change with workstations' capabilities. More importantly, file size in Excel sums to the total memory available to the solver to decrease the number of cells on which operations can be applied. While the above limits might seem practical for small-scale proteomics experiments, they quickly become too taxing for larger data files (*e.g.*, larger proteomics experiments, scRNA-seq data). Conversely, Matrisome AnalyzeR can handle middle to large-sized data files, operating memory-efficient operations without breaking.
For example, the file hnscc.RDS presented to users as part of our new test file gallery (https://sites.google.com/uic.edu/matrisome/tools/matrisome-analyzer), is kept within the limits accepted by our app (a limit we enforce to ensure a more agile user experience, as the app can technically handle more than 1 GB upload per single user session) only by the compression operated by the .RDS format, and quickly scales up to double the size due to the de-sparsification of the data matrix and the operations run on it. Nevertheless, Matrisome AnalyzeR can annotate the file in a few seconds and analyze each of the 370 single cells within a few minutes.
4) Comparing the capabilities of VLOOKUP function and Matrisome AnalyzeR. Setting aside

© 2023. Published by The Company of Biologists under the terms of the Creative Commons Attribution License (https://creativecommons.org/licenses/by/4.0/).

the other points, a user trying to perform an analysis using the VLOOKUP function would still need to have local, and up-to-date, collections of annotated matrisome genes and proteins across their model organisms of choice and to have them open in Excel at the same time as the data to be compared to. Provided that the memory space suffices, the result would be a set operation (how many elements of list A are also in list B) or, as stated by the reviewer, "compare two lists". This, however, is not what Matrisome AnalyzeR does. Rather, Matrisome AnalyzeR transfers annotations (matrisome divisions and categories, as well as now GO:CC ontologies) from a knowledgebase onto user data, enriching them without changing their structure and allowing their further use. With the "annotation and analysis" workflow, Matrisome AnalyzeR also operates column-wise summations of all numerical data available in the user-uploaded data, employing several efficient search and conversion strategies to handle mixed-type data such as those sporting the "%" symbol. Importantly, our web tool can perform data manipulation, plotting and analysis (if requested) in a matter of milliseconds to seconds for most files, up to a few minutes for very large files whose size and format is not compatible with Excel operations.
5) Limited data visualization options. Data visualizations on the web app are intentionally kept to the minimum to offer an at-a-glance overview of the results. For more sophisticated visualizations, we refer to the dedicated Matrisome AnalyzeR package, which offers relational (Sankey or alluvial) and polar plots which are not otherwise available in Excel.
Finally, we would like to stress that our manuscript not only describes one web tool but also provides a full R package, built to integrate seamlessly with any analytical pipeline in R and offering enhanced visualization facilities.

Reviewer 1 Comments for the Author:

The problem is that the vast majority of researcher will not only want to check whether their list of genes contains extracellular matrix proteins, and therefore will find more useful a general tool to indicate enrichment of any type of gene. This is commonly done with GO enrichment tools, as GO annotation of gene product function is an active and ongoing project for these organisms.
> Matrisome AnalyzeR is evidently a more specialized product and was not primarily developed to perform enrichment analyses. It serves a different purpose and – likely – a different community. In fact, it is part of an ecosystem of tools and resources (the Matrisome Project, https ://s i tes .google. com/u ic.edu /ma tris om e/) dedicated entirely to ECM research.

With respect to GO enrichment, we would like to point that one of the reasons why matrisome annotations were initially developed was to overcome the limitations of ECM annotations in GO, which are either too general and poorly informative or too granular - when not erroneous - for a more specialized community (see Naba *et al.*, Mol Cell Prot, 2012 and Naba *et al.*, Matrix Biol, 2012).
To illustrate the differences between matrisome and the "ECM" annotations provided by GO, we attempted to annotate one of the test input files (example 1) provided with Matrisome AnalyzeR. To do so, we first downloaded the latest annotation tables for the different model organisms from Gene Ontology (w w w.geneontology.org). Within the GO hierarchy, we analyzed both the uppermost available ECM terms from the Cellular Component (CC) ontologies ("GO:0005576" and "GO:0044421", the latter obsolete but still present within the annotation files) and the more downstream terms "GO:0005578" and "GO:0031012" (*see Table 1*).

Table 1. Number of annotations in the GO:CC class for the different model organisms.

Results for "GO:0005576" and "GO:0044421"			
	Total annotations	ECM annotations	Non-ECM annotations
Homo sapiens	43003	3909	39094
Mus musculus	67248	3566	63682
Danio rerio	51398	2013	49385
Drosophila	30401	1000	29401
C. elegans	29485	856	28629

Results for "GO:0005578" and "GO: 0031012"

© 2023. Published by The Company of Biologists under the terms of the Creative Commons Attribution License (https://creativecommons.org/licenses/by/4.0/).

	Total annotations	ECM annotations	Non-ECM annotations
Homo sapiens	39617	410	39207
Mus musculus	64190	489	63701
Danio rerio	49695	274	49421
Drosophila	29685	260	29425
C. elegans	28729	46	28683

As we noted in a past commentary (Naba *et al.*, Matrix Biol, 2012), a large subset of genes receives both ECM and non-ECM GO:CC annotations (*see Table 2*).

**Table 2. Number of genes with single (ECM *or* non-ECM) and double (ECM *and* non-ECM) annotations in the GO:CC class for the different model organisms.**		
Results for “GO:0005576” and “GO:0044421”		
	single annotations	double annotations
Homo Sapiens	35411	3796
Mus Musculus	60162	3543
Danio Rerio	47446	1976
Drosophila	28449	976
C. Elegans	27881	802
Results for “GO:0005578” and “GO: 0031012”		
Homo Sapiens	38797	410
Mus Musculus	63220	485
Danio Rerio	49149	273
Drosophila	29165	260
C. Elegans	28637	46

When transferring “GO:0005578” and “GO: 0031012” annotations to the Matrisome AnalyzeR example file, the differences with matrisome annotations and their limited performance become evident (*Table 3*).

**Table 3. Comparison between matrisome and GO:CC annotations in the Matrisome Analyzer test file.**							
GO:CC vs Annotated Matrisome Division							
	Core matrisome		Matrisome-associated			Non-matrisome	
GO:CC extracellular matrix & GO:CC not extracellular matrix	27		2			11	
GO:CC not extracellular matrix	36		25			495	
GO:CC vs Annotated Matrisome Category							
	Collagens	ECM-affiliated proteins	ECM Glycoproteins	ECM Regulators	Proteoglycans	Secreted Factors	Non matrisome
GO:CC extra cellular matrix & GO:CC not extracellular matrix	3	0	17	2	7	0	11
GO:CC not extracellular matrix	14	7	20	17	2	1	495

In the test file, every ECM protein was annotated as both ECM and non-ECM, clearly introducing a bias that would affect any subsequent hypergeometric test. It is important to note that “non matrisome” genes receive ECM GO:CC annotations in 11 out of 506 (~2%) cases. These “non matrisome ECM genes” are not part of the functional ECM defined as the structural scaffold providing support to cells, though they could be primarily located outside the cell in some cases - examples including *ADD1* (Alpha-adducin), chaperone proteins such as the CCT family members.

© 2023. Published by The Company of Biologists under the terms of the Creative Commons Attribution License (https://creativecommons.org/licenses/by/4.0/).

At the same time, also the opposite phenomenon ("matrisome non-ECM genes") is observed, often with spectacular inconsistencies. For example, among the collagens, only *COL14A1*, *COL5A2* and *COL6A3* match with the GO terms above - and only a wider search including the top-hierarchical term GO:0005576 recovers the rest of the collagens. In all these examples, matrisome annotations allow for better precision and more significant insights, avoiding biases and ontological inconsistencies introduced by the different GO annotations.
Despite these limitations, we have now integrated a larger set of GO:CC annotations (GO:0005576, "GO:0005578" and "GO: 0031012") for all the model organisms into the results from the new version of Matrisome AnalyzeR, to aid a more complete understanding and visualization of the results.

Relevant to this, there is no mention of how the ECM protein/gene lists will be updated to incorporate new discoveries; without this the lists will progressively go stale.
> We thank the reviewer for this relevant comment. Determining whether a protein belongs to the matrisome or not is based on the presence of specific protein features (signal peptide, specific domains, or motifs) identified via genome-wide *de-novo* sequence analysis (see Naba *et al.*, Mol Cell Prot, 2012; Gebauer and Naba, 2020). Thus, the inference of a protein belonging to the matrisome is robust for organisms whose genomes and proteomes are well-characterized and is independent of whether there is evidence for a protein to function in the ECM. As such, the approach is largely unaffected by new experimental discoveries. An exception to that was the discovery in the mid-2010s of kinases responsible for the phosphorylation of secreted and ECM proteins which prompted us to deploy new releases of the human and murine matrisome lists in 2014. Since 2014, we have not identified from our own experimental work or others' genes that we had not predicted to potentially encode proteins part of the ECM. We feel competent to comment on the topic, since we have been maintaining the only database of ECM proteomics datasets, MatrisomeDB since 2016. Of note, in the early days of matrisome research, we were often told that our list of predicted matrisome genes was too large and that we had "over-predicted" the matrisome, since attempts at profiling the matrisome of tissues only identified a small portion of *in-silico* predicted matrisome components (see Naba *et al.*, Matrix Biol, 2016 and Shao *et al.*, Nuc Acids Res, 2020). However, the latest release of MatrisomeDB revealed a near complete coverage of the *in-silico* predicted matrisome (Shao *et al.*, Nuc Acids Res, 2023), validating our *in-silico* predictions. As a side note, we would also like to point out that the lists are gene-centric and the use of gene identifiers is much more stable than the use of protein identifiers, which prevents the list from going stale. We have now described why the lists are somewhat "resistant" to staleness (*see page 3 of the revised manuscript*).

I would urge the authors to work with HGNC, Uniprot, and the model organism databases to capture the information of the curated ECM gene lists they have produced via GO annotation and Gene Groups, as then the information will be accessible via diverse GO enrichment tools and updated with new research.
> We thank the review for this suggestion and whole-heartedly agree. Over the past decade and a half, we have always had an eye toward developing tools that are broadly accessible and have been working with the developers of other databases for many years. For example, as stated in the manuscript, our publication of the *Drosophila* matrisome was concomitant with the deployment of the *Drosophila* matrisome within the "Gene Groups" section of the 2019-04 release of FlyBase. We have also had a long-standing collaboration with the team of Dr. Jill Mesirov who developed and maintains the Molecular Signature Database (MSigDB), which is an integral part of the Gene Set Enrichment Analysis (GSEA) pipeline. The list of human matrisome genes has been incorporated to MSigDB since 2016, and the list of murine matrisome genes will be deployed in the next release of the mouse MSigDB scheduled in September 2023. Gene Ontology is also now listing the term "matrisome" as a synonym for "extracellular matrix" but has not implemented the additional matrisome classification yet. We are hopefully that the nomenclatures we have proposed over a decade ago and that have been largely adopted now, will have an even broader reach and adoption with the deployment of user-friendly tools such as Matrisome AnalyzeR.

For these reasons, I doubt that there will be extensive use of this tool.

> Since launching the Matrisome AnalyzeR page on the Matrisome Project website (https ://s i tes .google. com/u ic.edu /ma tris ome /tools / ma tris ome - an alyz er), traffic monitoring via Google Analytics shows that we have accrued more than 480 visits from 260 users.

© 2023. Published by The Company of Biologists under the terms of the Creative Commons Attribution License (https://creativecommons.org/licenses/by/4.0/).

In shinyapp.io (the host for the Matrisome AnalyzeR web app), each app instance spawns at least one “worker”, which serves up to 50 connected users at low workload before spawning a new worker process. We strictly enforce a no-tracking, zero-data retention policy, and thus do not log users, but a simple calculus from the “worker processes” graph indicates that, our app has registered anywhere from 2 (approx. 10% or 0.1 of 25) to 22 (approx. 90% or 0.9 of 25) more intensive users per day. Furthermore, as the memory use is largely below the absolute app threshold (8 GB) and the number of workers has never reached beyond one, we surmise the app load has been within the limits of a single worker (50 less demanding users), doubling the figures calculated above.

***Rev Figure 1*. Traffic monitoring of the Matrisome AnalyzeR page of the Matrisome Project website for the 4/20/2023 - 7/20/2023 period (source: Google Analytics).**

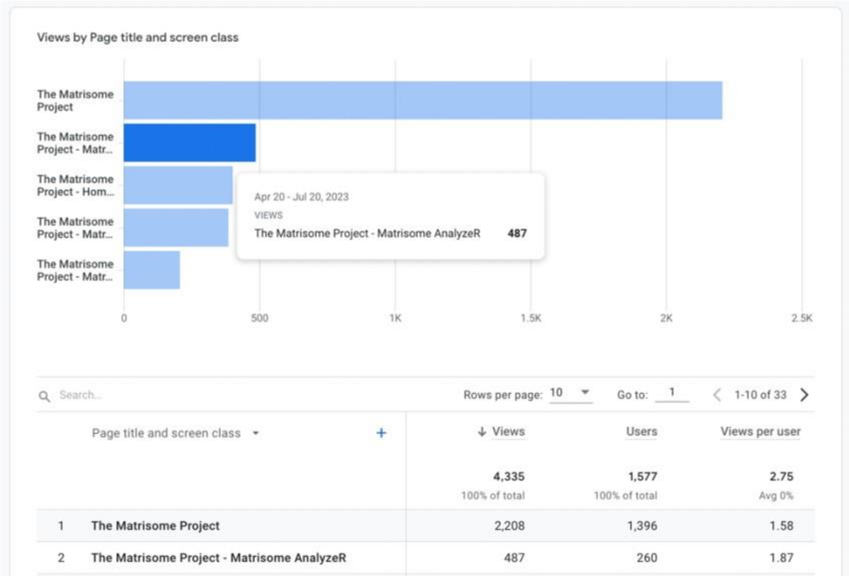

***Rev Figure 2*. App usage in the past tracking period according to Shinyapps.io**

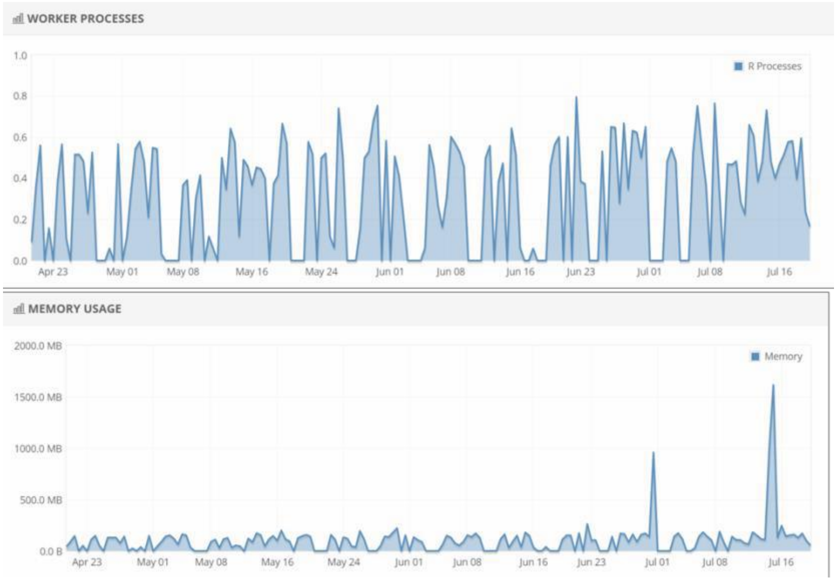

Reviewer 2

Advance Summary and Potential Significance to

© 2023. Published by The Company of Biologists under the terms of the Creative Commons Attribution License (https://creativecommons.org/licenses/by/4.0/).

Field:

This work has the potential to be a successful tool for ECM research. The work is interesting and could be resourceful for the ECM community.
> We thank the reviewer for this positive comment.

Reviewer 2 Comments for the Author:

However, there are certain points that needed to be addressed by the authors:

1. What kind of MS quantitation would be utilised for input file? IS it spectral count or LFQ or MS1 based quant? It is not clear. This is important that in general, researchers uses many different open-source and vendor specific search engine based methods for quantitative Mass-Spectrometry.
> Matrisome AnalyzeR has been developed to be versatile and handle not only mass spectrometry datasets but also other types of data such as RNA-Seq data or even genomic data. We have now emphasized this (*see page 3 of the revised manuscript*). As such, users can input any quantitative metrics they wish. For example, in the test file originally provided (now called "Example 1"), the metrics used are total spectral counts, unique spectral counts, unique peptide numbers. But the tool can handle **any** numerical value including total precursor ion intensities for mass spectrometry data, or number of reads for RNA-Seq datasets. We have now stated this more explicitly (*see page 5 of the revised manuscript*)

2. It would be great if the authors can at least integrate the output files from the most popularly used quant tools such as Maxquant and Skyline (open source).
> We thank the reviewer for this suggestion. We have now expanded the functionalities of the Matrisome AnalyzeR app to handle any tabular file format, including generated in MaxQuant, as well as raw skyline (.sky) files and R Data Serialization (.rds) files. We are also providing a new gallery of example files, accessible via the app and the Matrisome AnalyzeR page of the Matrisome Project website (https://sites.google.com/uic.edu/matrisome/tools/matrisome-analyzer) that users can use to familiarize themselves with Matrisome AnalyzeR before running their own datasets (*see pages 4 and 9 of the revised manuscript and revised Figure 1A*).

3. Authors should include in the input template regarding the quant criteria based on at least 2 or more unique peptides. Because, there could be multiple 1 peptide hits. For example, in the provided example data sheet- there are many isoforms of T-complex protein 1. How do the authors ensure the individual isoform protein group ID?
> What the reviewer is referring to is pre-analysis thresholding. Matrisome AnalyzeR is not a substitute for software performing mass-spectrometry database searching and analysis (grouping), *etc*. Users should perform those steps (*i.e.*, setting thresholds in terms of acceptable peptide and protein FDRs, exclusion of proteins detected with less than x number of peptides, determination of protein grouping method and whether to include share peptides or not) **prior** to inputting their data file to Matrisome AnalyzeR. We have now stated this more clearly in the manuscript (*see page 4 of the revised manuscript*).

4. It is possible that, the data was acquired by a rigorous biochemical enrichment of ECM (following decellularization) followed by MS analysis still a number of cytoskeletal proteins were identified. It has been shown that cytoskeleton proteins could actually be connected with the ECM and most of the times they are indeed enriched with ECM proteins (example- Suleiman et.al eLife-Jeff Miner's work). How do the authors would annotate them? Comments and relevant modification on this aspect would be appreciated.
> This is a very interesting point. Many cytoskeletal proteins are indeed connected via cell-surface receptors to the ECM, and this can explain partially why cytoskeletal proteins may be detected in decellularized samples. It is also worth keeping in mind that, like ECM proteins, many cytoskeletal proteins are also intrinsically highly insoluble and their presence in a decellularized sample may simply results from the difficulty of extracting them (independently on their interactions with ECM proteins). To help users identify the nature of non-matrisome components present in their samples, we have added Gene Ontology annotations on Cellular Components (*see page 5 of the revised manuscript and revised Supplementary Table S2*).

© 2023. Published by The Company of Biologists under the terms of the Creative Commons Attribution License (https://creativecommons.org/licenses/by/4.0/).

5. Could the authors built in a plug-in tool to prepare an abundance curve of the ECM proteins to estimate the distribution pattern?
> At the moment, Matrisome AnalyzeR does not intend to compute individual-protein-level (or individual- RNA-level) abundances. Matrisome AnalyzeR is designed to provide an overview of the molecular matrisome landscape of their samples.

6. If the authors aims to analyze based on relative quant then statistical tests should be incorporated with FDR correction option.

> Statistical analyses are highly dependent on data types, experimental designs, and the overall questions experimenters are posing. It is thus difficult to predict what kind of analyses users will want to perform. Hence, at the moment, Matrisome AnalyzeR is not intended to perform statistical analyses. Rather, we are encouraging users to plug the datasets annotated using the "Annotate" function of Matrisome AnalyzeR into their own analytical pipeline. However, as we have done for other tools, we developed like MatrisomeDB, we intend to seek feedback from users and if recurrent suggestions arise, we will consider further expanding the functionalities of Matrisome AnalyzeR.

Second decision letter

MS ID#: JOCES/2023/261255

MS TITLE: Matrisome AnalyzeR: A suite of tools to annotate and quantify ECM molecules in big datasets across organisms

AUTHORS: Petar B Petrov, James M Considine, Valerio Izzi, and Alexandra Naba
ARTICLE TYPE: Tools and Resources

I am happy to tell you that your manuscript has been accepted for publication in Journal of Cell Science, pending standard ethics checks.

Reviewer 1

Advance summary and potential significance to field

The authors have done a good job addressing the reviewers comments.

Comments for the author

The authors have done a good job addressing the reviewers comments.

Reviewer 2

Advance summary and potential significance to field

Accepted

Comments for the author

Accepted

© 2023. Published by The Company of Biologists under the terms of the Creative Commons Attribution License (https://creativecommons.org/licenses/by/4.0/).
